# Accelerometer based assessment of daily physical activity and sedentary time in adolescents with idiopathic scoliosis

**DOI:** 10.1371/journal.pone.0238181

**Published:** 2020-09-02

**Authors:** Swati Chopra, A. Noelle Larson, Kenton R. Kaufman, Todd A. Milbrandt

**Affiliations:** 1 Motion Analysis Laboratory, Mayo Clinic, Rochester, MN, United States of America; 2 Leeds Institute of Rheumatic and Musculoskeletal Medicine, University of Leeds, Leeds, United Kingdom; 3 Department of Orthopedic Surgery, Mayo Clinic, Rochester, MN, United States of America; Linneaus University, SWEDEN

## Abstract

**Background:**

Studies have shown a positive correlation between higher physical activity (PA) and health benefits. However, device-based assessment of PA and sedentary time (ST) in people with adolescent idiopathic scoliosis (AIS) has not been deeply investigated.

**Objective:**

Analysis and comparison of weekend and weekdays PA and ST using multiple accelerometers in people with AIS with different curvature severity compared to healthy controls.

**Methods:**

24 participants with AIS divided into 2 groups of 12 with Cobb angles < 40° and > 40°, along with 12 age and BMI matched healthy controls. Daily PA and ST during four consecutive days were measured using four tri-axial accelerometers. Clinical functional assessment was performed using the scoliosis research society (SRS-22) questionnaire.

**Results:**

The combined weekend and weekdays average daily step count was found to be 22% and 29% lower in the AIS groups with Cobb angle < 40° and > 40°, respectively, compared to the controls. The average ST was also reported to be 5% and 7% higher in the AIS groups with Cobb angle < 40° and > 40°, respectively, compared to the controls. The reported differences were significant in the AIS group with higher Cobb angle (p≤0.05). No significant differences in PA or ST were reported between the AIS groups based on curvature severity.

**Conclusions:**

Decreased PA and increased ST observed in patients with AIS may have long term health implications and may play a role in the disease process. The device-based assessment of PA to understand potential benefits in clinical practice is recommended.

## 1. Introduction

Scoliosis is the abnormal three-dimensional curvature of the spine including hypokyphosis, rotation and coronal deformities. Scoliosis in children can be rapidly progressive during the growing years [1]. Approximately 90% of scoliosis cases fall under the category of adolescent idiopathic scoliosis (AIS) with no clear underlying syndromic or neuromuscular cause [1]. The prevalence rate of AIS is 0.47% - 5.2%, with a female to male ratio of 2:1, and the gender ratio gap is seen to increase with disease severity and age [1]. The degree of spinal curvature identified on a postero-anterior radiograph is measured by the Cobb angle [2], the angle subtended by the two most tilted vertebrae. AIS patients diagnosed with a Cobb angle > 20° require treatment based on factors including the location of the curvature of the spine, skeletal maturity, and age. Radiographic images are used as an indirect measure of skeletal maturity which is presented in terms of the Risser sign [3]. The Risser sign has 6 stages from 0 to 5, where Stage 0 represents no ossification in the iliac apophysis, and Stage 5 represents complete ossification and fusion of the iliac apophysis [3]. Curves between 25° to 40° in growing children are treated with spinal bracing to reduce the risk of curve progression [4]. Curves greater than 50° are thought to be progressive throughout life; thus, surgery is usually the treatment of choice for curves over 45° to 50° [5].

A longitudinal study including 4640 participants has reported that children performing regular, objectively measured, moderate to vigorous PA (MVPA) are 30% less likely to develop scoliosis [6]. An objectively measured increase in the ST in healthy adolescents has also been found to be negatively associated with bone health [7]. Another study has reported negative effect of low level of self-reported PA on psychological wellbeing in adolescents [8]. Prolonged relaxed sitting in healthy children has shown to increase trunk asymmetry [9]. In AIS patients, prolonged relaxed sitting led to an even greater trunk asymmetry due to the habitual leaning on one side depending on the curvature type [10]. Knowing the health complications related to scoliosis (musculoskeletal, psychological and respiratory conditions) which seem to progress with an increase in the scoliosis curvature [11–14] it is important to identify PA and ST as important factors in assessing health status of patients with AIS.

It is quite evident that participation in regular PA is important for patients with AIS for health benefits, including improved bone mineral density (BMD), strength, mobility, balance and controlled curve progression, thereby improving their quality of life [15–18]. To achieve this successfully, it is important to have a valid and reliable method of assessment of PA. This will provide a more in depth understanding of the reduced exercise capacity and the factors affecting ST in patients with AIS in order to help design optimal interventions. However, studies reporting objectively measured PA and ST are uncommon in patients with AIS. In fact, only one survey-based study has compared PA in patients with AIS and their peers without AIS using the International Physical Activity Questionnaire Short Form (IPAQ-SF). The study concluded that patients with AIS have similar PA levels as their peers without AIS [19]. Notably, the IPAQ-SF questionnaire has been shown to exaggerate PA levels compared to the device-based measurements [20].

This study, therefore, aims to assess the daily PA and ST in patients with AIS objectively, based on the severity of their scoliosis curvature, and compare their activity status with healthy age and BMI matched controls. The working hypothesis of the study is that patients who have AIS with different severity levels will have similar PA levels when compared with the age and BMI matched healthy controls.

## 2. Methods

### 2.1. Participants

During the years 2015–2018, 51 patients, who referred to the Mayo Clinic tertiary referral center for treatment from various socioeconomic backgrounds, were screened to participate in the study. Out of 51, 14 patients declined and 37 agreed to participate. This cross-sectional study included 24 (17F/7M) consecutive patients with AIS, and 12 age, BMI and gender matched controls without AIS. The 24 patients with AIS included 12 patients with Cobb angles < 40° and a Risser’s stage between 0–2 who were advised to have spinal bracing treatment, and 12 patients with Cobb angles > 40° and Risser’s stages of 4 and 5 who were advised to have spinal fusion (Table 1). The control group consisted of children who were referred due to acute injuries that did not impair their mobility and also fulfilled the inclusion criteria of no prior or existing spinal or lower extremity deformities. The study protocol was approved by the Institutional Review board. Informed consent was obtained from all study participants in the presence of their parents/ guardians.

**Table 1:** Demographics of participants.

Demographics	AIS Cobb angle < 40	AIS Cobb angle > 40	Controls
**Participants**	12	12	12
**Gender**	9F/ 3M	8F/ 4M	9F/3M
**Age (years)**	12.4 (1.1) ^b^	14.3 (1.4) ^b^	13 (5)
BMI (kg/m^2^)	17.8 (2.6) ^b^	22.2 (4.1) ^b^	19.65 (3.1)
**Average of maximum Cobb angle**	27 (4) ^b^	57 (9) ^b^	-
**Risser’s stage**	0–2^b^	4–5^b^	-

^a^P<0.05 difference compare to controls

^b^P<0.05 difference between AIS groups

### 2.2. Assessment methods

Assessment was performed before any treatment was started. The subjective assessment of health, based on the patient reported outcome measure (PROM), was carried out using the commonly used SRS-22 questionnaire [21]. Scoring is based on the set of 22 questions related to pain, self-image, mental health, function and satisfaction. The final score gives the combined outcome of all 5 subsections and a higher score represents a better outcome. Although the validity and reliability of the SRS-22 questionnaire has only been proven among adults [22, 23], it is still widely used in studies assessing the outcome of treatments in AIS [24–26]. This study utilized the SRS-22 questionnaire to compare the functional, pain and general health status of AIS patients with different curvature severity, to identify any differences between the groups.

The Cobb angle and Risser sign were calculated from the postero-anterior radiographic images. Radiographic imaging was not performed for the healthy controls. The PA of® all participants was assessed objectively based on the time spent being active or inactive. Field-based activity data was collected using four miniature tri-axial accelerometers (Actigraph GT3X+, Pensacola, FL, USA). All four activity monitor units (AMU) were calibrated to record +1g, 0g, -1g and were synchronized to each other based on a previously validated protocol [27].

The AMU were placed on both ankles, on the right thigh and at waist level, either over or under clothes. Activity data was collected at a frequency of 100 Hz. Based on the previous studies, device-based assessment of daily PA in children for least 10 hours/day and 4 days of monitoring including weekend and weekdays, is considered reliable [28–30]. In this study, all participants, including the controls, were asked to wear the sensors for 4 consecutive days (2 weekdays and 2 weekend days) from the time they were out of bed and bathed in the morning until the time they returned to bed at night. Participants were asked to remove the sensors during any water activities e.g. bathing/ swimming.

### 2.3. Signal processing

Analysis, calibration and processing of the AMU data was carried out using the MATLAB R2015b (Version 7.11.0, Mathworks, MA, USA). A validated algorithm-based analysis of AMU data was used to detect movement and postural transitions during active and inactive periods [27]. More specifically, the waist AMU signals were used to differentiate between static and dynamic activity [27]. Dynamic activity is established by calculating when the signal magnitude areas (SMA) exceed a threshold of 0.135 g continuously for a period of 1s. SMA below the 0.135 g threshold were further analyzed via the application of a continuous wavelet transform using a Daubechies 4 Mother Wavelet [27]. Data, which fell within a range of 0.1–2.0 Hz and also exceeded a scaling threshold of 1.5 over each second, were also identified as dynamic activity [27]. SMA between 0.135 g and 0.8 g were considered light physical activity (LPA), including walking, while SMA exceeding 0.8 g were considered as MVPA, including jogging and running [31].

A step detection algorithm analyzed the ankle AMU data to identify heel strikes from the anterio-posterior signal, further differentiating into walking and jogging steps [27]. Finally, a postural orientation algorithm analyzed the thigh and waist AMU data to estimate the torso angle and differentiate between different postures (standing, sitting and lying down) as well as the transition between them [27]. A postural transition is recorded if a different position (standing, sitting or lying) is identified prior to and following a 2 second time-frame. Postural transitions are counted as active time periods [27].

### 2.4. Statistical analyses

AMU data and SRS-22 scores are presented as the group mean. The z score is assessed to test the distribution of data in each group [32]. The z score results showed normal distribution in our data set. Firstly, to compare PA and ST between the controls and the AIS groups, an analysis of variance (ANOVA) was performed to analyze the differences between the group means. The post hoc comparisons were performed using the Dunnett’s correction to control the Family-Wise Error Rate [33]. The effect size (d) was calculated to study the power of the analysis. Secondly, the differences in PA and ST were compared between the AIS groups with < 40° and > 40° Cobb angles after adjusting for age and BMI by using an analysis of covariance (ANCOVA). All the outcomes were presented as a mean with standard deviation (SD). The significance level for all statistical tests was set at p≤0.05.

## 3. Results

The recorded wear time of the AMU for all participants over weekdays and weekends was an average of 13.5±1.7 hours/day. The patient reported SRS-22 questionnaires, in both AIS groups, showed a comparable health status (Table 2).

**Table 2:** SRS-22 outcome in means (SD).

Group AIS	Total	Function	Pain	Self-image	Mental health	Satisfaction
**Cobb angle < 40**	4.18 (0.55)	4.38 (0.42)	4.43 (0.85)	3.96 (0.78)	4.35 (0.58)	3.29 (0.58)
**Cobb angle > 40**	4.1 (0.49)	4.4 (0.24)	4.24 (0.53)	3.71 (0.83)	4.22 (0.72)	3.86 (0.90)

The PA and ST outcomes during the two weekdays is given in (Table 3). The AIS group with Cobb angle > 40° reported a significantly less percentage of time spent physically active (p<0.02, d = 0.87), especially time spent in LPA (p<0.003, d = 1.2), as well as a significantly low daily step count (p = 0.02, d = 0.94) compared to the controls. The percentage of ST is also found to be significantly high (p = 0.02, d = 0.87) compared to the controls. While, the AIS group with Cobb angle < 40° also showed increased inactivity compared to the healthy controls, the difference was not significant. The only significant difference was reported in the percentage of time spent in LPA (p = 0.005, d = 1.66).

**Table 3:** Activity and sedentary behavior during weekdays. Mean (SD).

Groups	AIS Cobb angle < 40 (n = 12)	AIS Cobb angle > 40 (n = 12)	Controls (n = 12)	ANOVA *p value*	Dunnett’s *D value*
**% of time spent sedentary**	81.7 (4.9)	82.6 (6.2) ^a^	77.06 (5.1)	0.038	5.1
**% of time spent standing**	12.6 (5.4)	10.9 (5.4) ^a^	15.98 (4.5)	0.07	4.81
**% of time spent sitting**	36.9 (12.2)	36.7 (13.6)	34.5 (9.5)	0.8	11.05
**% of time spent lying**	29.16 (13.2)	34.0 (16.6)	23.7 (6.5)	0.17	11.84
**% of time spent active**	18.3 (4.9)	18.16 (5.8) ^a^	22.9 (5.1)	0.038	5.1
**% of time spent in LPA**	10.4 (2.8) ^a^	11.4 (3.2) ^a^	15.5 (4.4)	0.00067	3.02
**% of time spent in MVPA**	0.69 (0.5)	0.23 (0.24)	0.66 (0.59)	0.04	0.44
**Daily steps**	8 188 (2606)	7470 (3012) ^a^	10301 (2367)	0.037	2511

^a^ Represents significant difference (p<0.05) compared with the controls. The difference of mean between the controls and the AIS groups should be larger than the D value for the difference to be significant

The PA and ST outcomes during the two weekend days is given in (Table 4). Reduced PA is reported in all three groups. However, a significant difference was only reported in percentage of time spent in MVPA in the AIS group with Cobb angle > 40°, compared to the controls (p = 0.005, d = 1.66).

**Table 4:** Activity and sedentary behavior during weekend days. Mean (SD).

Groups	AIS Cobb angle < 40 (n = 12)	AIS Cobb angle > 40 (n = 12)	Controls (n = 12)	ANOVA *p value*	Dunnett’s *D value*
**% of time spent sedentary**	81.9 (4.6)	81.6 (7.3)	77.8 (9.8)	0.35	7.07
**% of time spent standing**	11.7 (5.7)	12.8 (5.6)	15.7 (6.2)	0.25	5.5
**% of time spent sitting**	36.9 (9.7)	30.5 (12.5)	28.9 (13.3)	0.24	11.16
**% of time spent lying**	32.0 (10.7)	34.6 (17.5)	33.9 (14.0)	0.9	13.39
**% of time spent active**	18.1(4.6)	18.4 (7.3)	22.1 (9.8)	0.35	7.07
**% of time spent in LPA**	9.8 (2.9)	10.5 (4.9)	14.0 (5.8)	0.07	4.39
**% of time spent in MVPA**	0.59 (0.5)	0.18 (0.17) ^a^	0.65 (0.56)	0.04	0.43
**Daily steps**	6666 (2230)	6085 (3364)	8800 (3925)	0.13	3026

^a^ Represents significant difference (p<0.05) compared with the controls. The difference of mean between the controls and the AIS groups should be larger than the D value for the difference to be significant.

**Fig 1:**
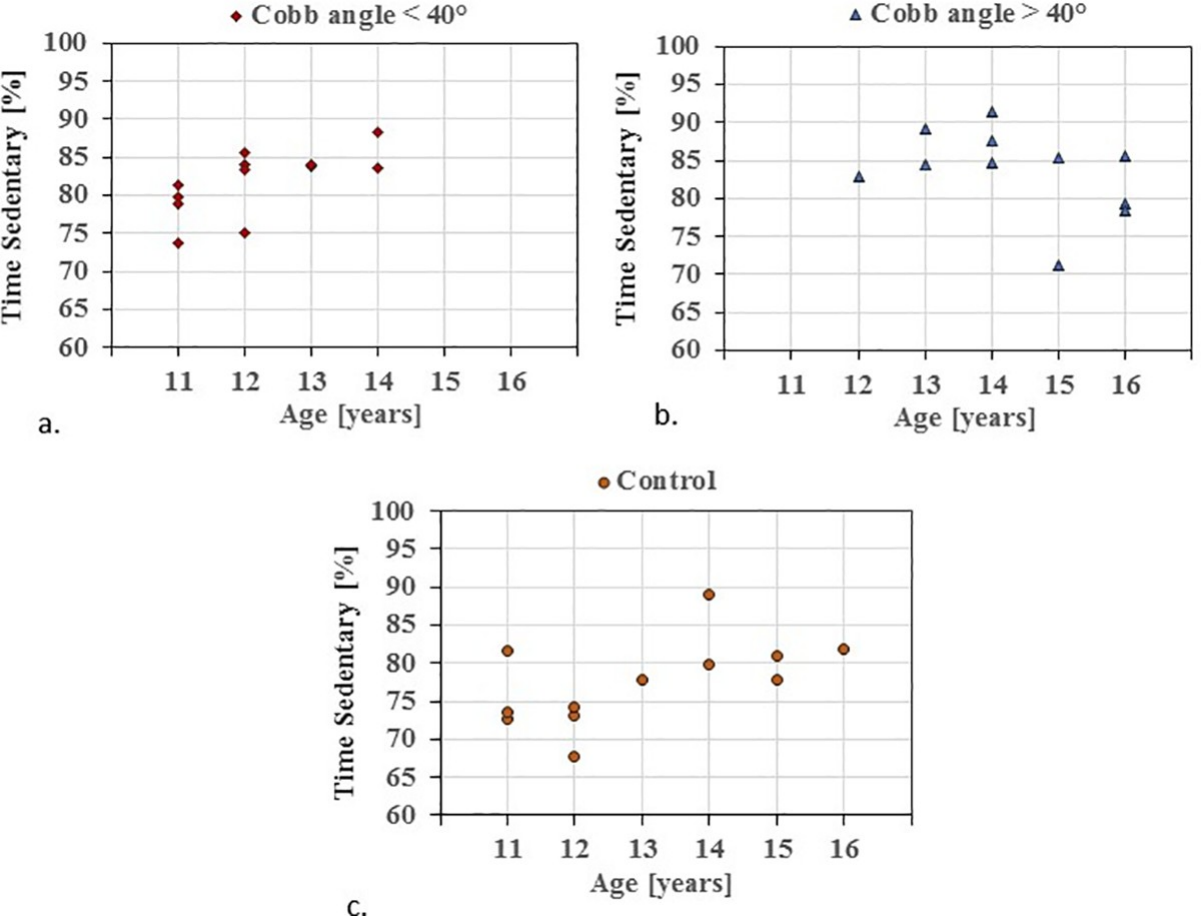
Individual data plot representing the average sedentary time (%) in relation to age in (a.) AIS Cobb angle <40°, (b.) AIS Cobb angle >40°and (c.) Controls.

The ANCOVA comparison between the two AIS groups, after adjusting age and BMI, reported no significant differences in PA or ST parameters. Fig 1 shows the relation between ST in % and age of the participants, in all three groups.

## 4. Discussion

This study presents a device-based assessment of the daily PA levels and ST in patients with AIS. The quantification of PA levels was performed using accelerometers, applying validated methods [34]. A lower PA is observed in patients with AIS, irrespective of the curvature severity, compared to the controls. Therefore, the outcome of this study rejects the hypothesis that patients with AIS, irrespective of curvature severity, are as equally active as their peers without AIS.

Previous investigations have revealed 10,000 to 16,000 steps/day to be optimal for health benefits, which represents approximately ≥60 mins/day of MVPA in healthy adolescents [35, 36]. In our study, the patients with AIS, irrespective of the curvature severity, do not seem to achieve the recommended step counts, both during weekdays and weekends. The PA outcome aligns with the previous study assessing daily step count using a pedometer in AIS patients with Cobb angle <50° reporting lesser daily steps than the recommended for health benefits [37]. In AIS patients, during walking, a 30% increase in energy cost and a 20% -30% decline in muscle efficacy has been identified [38]. This could be one of the reasons behind the decreased number of daily steps and lower percentage of LPA reported in our cohorts with AIS, irrespective of curvature severity. Although, this not being an issue for healthy adolescents, our control group participants failed to achieve the recommended daily step count of 10000 steps during weekends. Previous studies, on healthy adolescents, reports that after school activity accumulates greater amount of PA [39, 40]. Therefore outcome of this study further confirms the importance of after school physical activity in all adolescents. Notably, the ST reported in this study also falls within the interquartile range of the ST reported in a previous study for healthy participants between ages 10 to 11 years [41]. Although, the average ST reported in this study, for the three groups, are on the higher end of the interquartile range, this could be due to the age range of the participants in our study (ages 11 to 16 years), knowing that ST seems to increase with age in adolescents [42].

Based on the above discussion, there is a need for a recommendation that patients with AIS, irrespective of curvature severity, are encouraged to participate in sports and regular PA. Unfortunately, most of the studies assessing the effect of therapeutic spinal exercises in patients with AIS only include mild severity cases [43, 44]. A recent meta-analysis based on the outcome of 15 studies has reported that the effect of therapeutic Schroth exercises is more beneficial for Cobb angles < 30° [45]. Furthermore, in idiopathic scoliosis, participation in sports which can strain the back leading to increased axial loading and hyperlordosis are not indicated if there is an underlying problem of back pain. It is also suggested that sports recommendation should be individualized in patients with curvature severity [46]. This could be one of the many reasons why patients with AIS are not encouraged to participate in sporting activities.

However, to achieve the health benefits, an increase in daily PA and a reduction in ST in patients with AIS are crucial. A study has shown the need to assess PA and ST in children objectively, to understand the factors affecting activity levels in order to develop tailored physical intervention [47]. There is also a clear need for health education regarding the risks and benefits of structured PA and the importance of an increase in PA and a reduction in ST for patients and their families in managing AIS. We suggest a device-based assessment of daily activity, similar to that carried out in this study, would provide accurate PA information, which in the future could help develop patient-specific PA guidelines, potentially preventing osteopenia and reducing other health problems related to AIS, perhaps even controlling progression of scoliosis.

The strength of the study follows the use of a validated AMU system capable of monitoring activity levels continuously over an entire day. PA levels were monitored for four consecutive days, including weekend and weekdays, to take into account the inherent differences. Furthermore, the utilized multi-sensor accelerometer array allows one to differentiate, not only between activity and inactivity, but also between different static position and postural transitions. The small study size is however a study limitation, though the significant differences showed an acceptable effect size. Another limitation of the study is the missing standardized method of grading leisure and sporting activities at the baseline. 90% of AIS patients in the study self-reported participation in regular organized PA. Based on the results of the study, this appears to be an overestimation.

## 5. Conclusion

The findings of our study illustrate the differences in PA and ST between patients with AIS and the healthy controls. Notably, curvature severity was not shown to have much effect on PA or ST when AIS groups with mild and severe Cobb angles were compared. The study suggests the use of device-based PA monitoring for more descriptive information on PA and ST in patients with AIS. In terms of improving treatment prognosis in AIS, it is important to include PA and exercise recommendations, based on the severity of the spinal curvature, in clinical practice.
